# High dietary non-starch polysaccharides detrimental to nutrient digestibility, digestive enzyme activity, growth performance, and intestinal morphology in largemouth bass, *Micropterus salmoides*

**DOI:** 10.3389/fnut.2022.1015371

**Published:** 2022-10-26

**Authors:** Yu Liu, Jiongting Fan, Huajing Huang, Hang Zhou, Yixiong Cao, Yumeng Zhang, Wen Jiang, Wei Zhang, Junming Deng, Beiping Tan

**Affiliations:** ^1^College of Fisheries, Guangdong Ocean University, Zhanjiang, China; ^2^Aquatic Animals Precision Nutrition and High Efficiency Feed Engineering Research Centre of Guangdong Province, Zhanjiang, China; ^3^Key Laboratory of Aquatic, Livestock and Poultry Feed Science and Technology in South China, Ministry of Agriculture, Zhanjiang, China

**Keywords:** non-starch polysaccharides, largemouth bass, growth performance, apparent nutrient digestibility, intestinal morphology

## Abstract

An 8-weeks feeding trial was carried out to evaluate the effects of different levels of dietary non-starch polysaccharide on the growth, apparent nutrient digestibility, intestinal development, and morphology of largemouth bass (*Micropterus salmoides*). Seven isoproteic and isolipidic experimental diets were formulated (crude protein 47.00%, crude lipid 12.50%), containing 0, 3, 6, 9, 12, 15, and 18% non-starch polysaccharides (NSPs) (named Control, NSPs3, NSPs6, NSPs9, NSPs12, NSPs15, and NSPs18), respectively. Dietary inclusion of NSPs below 9% showed no negative impacts on fish growth and feed utilization efficiency, whereas dietary NSPs inclusion level above 9% decreased weight gain rate, specific growth rate, protein efficiency, protein deposition rate, apparent digestibility of dry matter and protein, and were accompanied by a reduction in intestinal protease, Na^+^/K^+^-ATPase and alkaline phosphatase activity and an increase in feed intake and feed coefficient. The activity of lipase was significantly decreased when dietary inclusion of 15 and 18% NSPs. Moreover, the lipid deposition rate and the apparent digestibility of lipids were significantly decreased since dietary inclusion of 9% NSPs. Dietary inclusion of NSPs above 12% significantly up-regulated intestinal GLP-2 gene’s expression, and was accompanied by significant changes in hindgut morphology, including increases in villus length and width, muscularis thickness and number of goblet cell, as well as a decrease in crypt depth. Additionally, dietary inclusion of NSPs above 3% significantly increased intestinal length index, and the viserosomatic index was significantly increased when dietary NSPs exceeded 15%. The linear regression analysis based on weight gain rate and feed coefficient showed that the appropriate dietary NSPs level of juvenile largemouth bass should not above 5.51%. In conclusion, high dietary NSPs adversely affects digestive enzyme activity and intestinal morphology, which in turn reduced the apparent digestibility of dietary nutrients and growth of juvenile largemouth bass.

## Introduction

Non-starch polysaccharides (NSPs) are the main components of plant cell walls, including cellulose, hemicellulose, and pectin, and are highly abundant in plant-based raw materials (1). The NSPs content of plant-based raw materials commonly used in aquafeed production has been reported to be as high as 22.7–50.5% (2). In recent years, global fishmeal production has been unable to meet the rising demand for fishmeal in the feed industry, resulting in increasingly high fishmeal prices (3, 4). In this situation, a large amount of plant-based ingredients was used as substitutes for fishmeal in aquafeed production in order to control feed costs (5, 6), and ultimately leading to a dramatic increase in the content of aquafeed NSPs. However, due to the lack of endogenous NSPs degrading enzymes (e.g., cellulases, β-xylanases, and β-glucanases) in monogastric animals (including fish), NSPs are usually not directly utilized by fish and remain in the digestive tract with varying degrees of physiological effects (1). Several studies have shown that dietary supplementation of 5% NSPs can effectively enhance the protein efficiency and growth of aquatic animals (7), but dietary supplementation with NSPs above 8% significantly inhibits the digestion and absorption of feed nutrients and growth performance of aquatic animals (8, 9), and may even cause functional damage or lesions in metabolic organs, resulting in metabolic disorders (2, 10–12). However, dietary fiber consisting of dietary NSP is also believed to be beneficial in helping fish resist pathogenic microbial infections and regulate metabolic and immune activities (13, 14). To sum up, the beneficial or detrimental physiological effects of dietary NSPs on fish may be closely related to their levels, and the results of available studies are inconsistent.

The physicochemical properties of dietary NSPs can drastically change the physicochemical properties of chyme, for instance, NSP will expand the volume of chyme after absorbing water, the viscosity of NSPs can lead to an increase in the viscosity of chyme, and some fermentable NSPs can increase the fermentability of chyme, etc. (1). Changes in the physicochemical properties of chyme may affect their flow rate in the intestine and the intensity of their friction with the intestine, and may produce different metabolites through fermentation, which in turn regulate the development and physiological state of the intestine. In pigs and poultry, chronic ingestion of NSPs led to an increase in the size and length of the digestive organs and caused an increase in the width of the jejunal and ileal villus and the depth of the crypt (15, 16). Similar reports were found in fish, dietary supplementation with NSPs (guar gum) significantly increased the gastrointestinal weight of African catfish (17), and dietary supplementation with 4% soy fiber significantly increased the length and number of hindgut villus and muscularis thickness but significantly decreased the width of hindgut villus and the number of goblet cells in largemouth bass, while increasing the soy fiber content to 8% significantly decreased the length of hindgut villus (18). Moreover, dietary fiber also dramatically altered the structural morphology of the intestinal tracts of larval cobia (*Rachycentron canadum*), gilthead sea bream (*Sparus aurata*), zebrafish (*Danio rerio*), and Jian carp (*Cyprinus carpio* var. *jian*) (19–22). The intestine is the main place for fish to absorb feed nutrients, and the changes in its structure and morphology are closely related to the digestive and absorption functions and growth of fish (23). The alteration of the internal structural and morphology of the intestine by dietary NSPs inevitably affects the digestion and absorption of feed nutrients in fish. This may be the reason why dietary NSPs have been observed in some studies to inhibit the absorption of feed nutrients (8, 9, 24), but it has not been confirmed. Furthermore, there is still a lack of systematic studies on the effects of dietary NSPs on fish intestinal development, morphology, and nutrient digestion and absorption. In intensive farming conditions, in order to control the cost and quality of feed, a large amount of plant-based raw materials or binders (e.g., microcrystalline cellulose, carboxymethyl cellulose) are inevitably used in the design of feed formulations (14, 25), which means that aquatic animals will face the effects of dietary NSPs for a long time. Dietary NSPs may have more extreme effects on carnivorous fish relative to herbivorous and omnivorous fish, because carnivorous fish usually do not have NSPs in their natural diet and their ability to utilize NSPs is relatively lacking. Therefore, it is urgent to clarify the potential physiological effects of dietary NSPs on carnivorous fish and provide data support for the production of carnivorous fish feed. This study aimed to evaluate the effects of different levels of dietary non-starch polysaccharide on the growth, apparent nutrient digestibility, intestinal development, and morphology of largemouth bass.

## Materials and methods

### Ethics approval

The juvenile largemouth bass used in this study was approved by the Animal Research and Ethics Committee of Guangdong Ocean University, China. Additionally, all experiments were performed in strict compliance with its guidelines.

### Experimental diets

Seven isoproteic and isolipidic experimental diets were formulated (crude protein 47.00%, crude lipid 12.50%), containing 0, 3, 6, 9, 12, 15, and 18% NSPs (named C, NSPs3, NSPs6, NSPs9, NSPs12, NSPs15, and NSPs18), respectively. Dietary NSPs were formulated with purified cellulose, β-glucan, mannan, arabinoxylan, and pectin in proportions as described in a previous study (12). Briefly, each portion of NSPs consisted of 4.52% β-glucan, 5.16% mannan, 19.35% arabinoxylan, 32.26% cellulose, and 38.71% pectin together, which were manually mixed homogeneously by the gradual mixing method. All ingredients were ground into powder and passed through a 0.30 mm diameter sieve, then accurately weighed and mixed with a Hobart-type mixer (JS-14, Zhejiang Zhengtai Elecric Co., Ltd., China). The fish oil, soybean oil, and soybean lecithin are mixed to prepare a mixed oil, then mixed with the raw ingredients and kneaded evenly by hand, and then pure water is added to make a dough. The experimental feed was prepared by extruding the dough through a 2.0 mm diameter die using a double-helix extruder (F-75, South China University of Technology, China). Finally, the feeds were air-dried at room temperature and stored at −20°C until used. The ingredients and proximate compositions of experimental diets are shown in Table 1.

**TABLE 1:** Formulation and proximate composition of experimental diet (%).

Item	Group						
	Control	NSPs3	NSPs6	NSPs9	NSPs12	NSPs15	NSPs18
**Ingredients**							
Fish meal^a^	55.50	55.50	55.50	55.50	55.50	55.50	55.50
Casein	12.00	12.00	12.00	12.00	12.00	12.00	12.00
Gelatin	3.00	3.00	3.00	3.00	3.00	3.00	3.00
Fish oil^a^	4.20	4.20	4.20	4.20	4.20	4.20	4.20
Soy oil^a^	3.20	3.20	3.20	3.20	3.20	3.20	3.20
Soy lecithin^a^	1.00	1.00	1.00	1.00	1.00	1.00	1.00
Starch	18.00	15.00	12.00	9.00	6.00	3.00	0.00
NSPs^b^	0.00	3.00	6.00	9.00	12.00	15.00	18.00
Ca (H_2_PO_4_)_2_^c^	1.00	1.00	1.00	1.00	1.00	1.00	1.00
NaCl^d^	0.20	0.20	0.20	0.20	0.20	0.20	0.20
Choline chloride	0.30	0.30	0.30	0.30	0.30	0.30	0.30
Vitamin C	0.03	0.03	0.03	0.03	0.03	0.03	0.03
Vitamin and Mineral premix^c^	1.50	1.50	1.50	1.50	1.50	1.50	1.50
Ethoxyquin^a^	0.02	0.02	0.02	0.02	0.02	0.02	0.02
Yttrium (III) oxide^d^	0.05	0.05	0.05	0.05	0.05	0.05	0.05
**Proximate composition**							
Moisture	10.91	11.10	11.10	11.22	11.08	11.15	11.27
Crude protein	47.02	47.19	47.21	47.02	47.01	47.01	47.12
Crude lipid	12.51	12.50	12.48	12.51	12.47	12.53	12.50
Ash	10.42	10.79	10.47	10.55	10.54	10.50	10.72

^a^Supplied by Zhanjiang Haibao Feed Co., Ltd. (Zhanjiang, China). Fish meal: 65.81% crude protein, 7.69% crude lipid; Fish oil, Soy oil, and Soy lecithin: 100% crude lipid.

^b^Supplied by Kunming Saijie Biotechnology Co., Ltd. (Kunming, China). NSPs, composed by cellulose (purity > 99.5%), arabinoxylan (purity > 99.5%), β-glucan (purity > 99.5%), mannan (purity > 99.5%), and pectin (purity > 99.5%).

^c^Supplied by Shanghai Macklin Biochemical Co., Ltd. (purity > 99.99%; Shanghai, China).

^d^Supplied by Sinopharm Chemical Reagent Co., Ltd. (purity > 99.99%; Shanghai, China).

^e^Supplied by Qingdao Master Biotech (Qingdao, China).

### Fish management

Juvenile largemouth bass provided by the freshwater aquaculture base of Guangdong Ocean University. After the largemouth bass juveniles were fasted for 24 h, 1,120 individuals with healthy physique, no disease or injury, and an average weight of 6.00 ± 0.01 g were randomly selected as test fish. The test fish were randomly divided into seven groups, each group had four net cages, and each net cage had 40 experimental fish (the cage size was 1.2 m × 0.8 m × 1.0 m). During the breeding period, the water quality was kept fresh, the water temperature was maintained at 28–31°C, the dissolved oxygen was more than 6.0 mg/L, and the ammonia nitrogen was less than 0.02 mg/L. The feeding experiment lasted for 8 weeks, and the experimental fish were fed twice daily until they were apparently satiated (07:00 and 17:00). The feed consumption and the number of test fish mortalities in each cage were recorded.

### Digestibility trial

The digestibility trial was performed during the feeding trial. Yttrium trioxide (99.9%, Sinopharm Chemical Reagent Co., Ltd., Shanghai, China) was used as an indicator in the experimental diets. After the test fish were acclimated to the test diet for 2 weeks, the collection of feces in each cage was started. The feces at the bottom of the cages were collected with a 200-mesh brail every day after the test fish had been fed for 5–7 h. The formed feces were then selected, dried at 65°C for 6 h, and stored at –20°C for subsequent analysis. Fecal collection was continued for 6 weeks to ensure that the volume of fecal samples met the assay requirements (dry matter >10 g).

### Sample collection

The test fish were fasted 24 h at the end of the feeding trial, and then the fish in each net cage were weighed and counted to calculate the growth index. Before sampling, the test fish were anesthetized with 100 mg/L of MS-222 (Sigma, USA). Three fish were randomly selected from each cage and frozen at −20°C for whole-body composition analysis. Four fish were randomly selected from each cage, and the total length of the fish was measured, weighed, and then dissected; the visceral mass, liver and intestine were sequentially weighed, and the length of the intestine was measured. Specifically, intestinal weights were weighed after removal of mesenteric fat. Another two fish were randomly selected from each cage for dissection, and the intestines were taken out and placed in EP tubes with RNA letter, snap-frozen in liquid nitrogen and then stored at −80°C for subsequent analysis.

### Chemical analysis

The chemical composition analysis of the experimental diets, whole-body and feces refers to the standard method (26). The samples were dried at 105°C to constant weight to determine the moisture content. The crude protein was determined according to the Kjeldahl method (*N* × 6.25); crude lipid by the Soxhlet extraction method; crude ash by burning in a muffle furnace at 550°C for 16 h. The determination of yttrium content in feed and feces was performed by inductively coupled plasma mass spectrometry (Agilent Agilent 7500cx).

### Intestinal enzyme activity analysis

Intestinal protein concentration and amylase, lipase, protease, creatine kinase (CK), alkaline phosphatase (AKP) and Na^+^/K^+^-ATPase activities were determined by commercial kits (ELISA, Shanghai Enzyme Link Biotechnology Co., Ltd.), and the sample processing and determination process were strictly in accordance with the kit instructions.

### Morphological observation of hindgut

One fish was randomly selected from each cage for dissection, and the hindgut (1 cm) was taken out and fixed with 4% formaldehyde. After standing for 24 h, the tissue samples were dehydrated in a series of graded ethanol, and embedded in paraffin after dehydration. After the paraffin was solidified, the intestinal paraffin was cut into slices with a thickness of 5 μm with a microtome, followed by hematoxylin-eosin staining, and encapsulated to make H&E sections. The prepared sections were observed with a Nikon Ni-U microscope imaging system (Nikon Ni-U, Japan), and the intestinal villus height and width, crypt depth, muscularis thickness, and number of goblet cells were counted according to the method described in a previous study (27).

Another one fish was randomly selected from each cage in the control group, NSPS 6 and NSPS 18 groups for dissection, and the hindgut tissue was fixed with 2.5% glutaraldehyde. After 24 h of fixation, the tissues were transferred into phosphate-buffered saline containing 2% osmium tetroxide, and then the tissues were dehydrated with a series of graded ethanol. After dehydration, the tissue was embedded in epoxy resin 812 and then cut into ultrathin sections using an ultra-microtome (Leica EM UC7, Japan) for uranyl acetate and lead citrate staining. Finally, the morphology of intestinal microvilli and intestinal epithelial cells was observed using a transmission electron microscope (HITACHI HT7600, Japan).

### Real-time quantitative RT-PCR assay

Intestinal total RNA was extracted with Trizol Reagent kit (Quanjin Bio, Beijing, China), followed by 1.2% denatured agarose to detect the integrity of total RNA, and a spectrophotometer (NanoDrop^®^ ND-2000, Thermo, USA) to detect the purity and concentration of total RNA. Subsequently, the total RNA was used as a template to synthesize complementary DNA (cDNA) with a reverse transcription kit (Accurate Biology, China), and stored at −20°C until use. The primer sequences used for fluorescence quantification are shown in Table 2, and the primers were synthesized by Shanghai Sangon Biotech (Shanghai) Co., Ltd., China. The mRNA expression levels were detected using a high-throughput fluorescent quantitative PCR instrument (480II) (Light Cycler480II, Thermo) under a 10 μl SYBR^®^ Green Premix Pro Taq HS qPCR Kit II (Accurate Biology, China) reaction system. Each sample was assayed in four replicates, and melting curve analysis was performed after each reaction to check product specificity. The mRNA expression of all genes was calculated by the 2^–ΔΔCT^ method, and the mRNA expression of the target gene was normalized with the *Ef1α* mRNA expression of the control group as the standard.

**TABLE 2:** Primer sequences for real-time PCR.

Gene	Primer type	Sequence 5′–3′	E-value (%)
Ef1α	F^a^	TGCTGCTGGTGTTGGTGAGTT	97.99
	R^a^	TTCTGGCTGTAAGGGGGCTC	
GLP-2R	F	CTTCAAGAGTGCGATGTGC	97.96
	R	GCCATAGCCTGTTGGTTTACTG	
IGF-1	F	CCGAGCAACACTGGTACTGA	115.63
	R	GCTGAGAGTGAGGTTGACGA	

^a^F, forward primer; R, reverse primer. IGF1, insulin-like growth factor 1; GLP-2, glucagon-like peptide 2.

### Calculations

The formula used in the study is as follows.


$$\mathrm{Survival}\mathrm{rate}\;(\mathrm{SR},\%)=100\times \left(\frac{\mathrm{final}\mathrm{fish}\mathrm{number}}{\mathrm{initial}\mathrm{fish}\text{number}}\right)$$

$$\mathrm{Weight}\mathrm{gain}\mathrm{rate}(\mathrm{WGR},\%)\;=\frac{100\times \left(\mathrm{final}\mathrm{body}\mathrm{weight}-\mathrm{initial}\mathrm{body}\mathrm{weight}\right)}{\mathrm{initial}\mathrm{body}\mathrm{weight}}$$

Specificgrowthrate(SGR,%/d)

$$\;=\frac{100\times \left[\mathrm{Ln}\left(\mathrm{final}\mathrm{body}\mathrm{weight}\right)-\mathrm{Ln}\left(\mathrm{initial}\mathrm{body}\mathrm{weight}\right)\right]}{\text{days}}$$

$$\mathrm{Feed}\mathrm{intake}\;\left(\mathrm{FI},\%\mathrm{body}\frac{\text{weight}}{\text{d}}\right)\;=100\times \frac{2\times \mathrm{feed}\mathrm{consumption}\times \text{days}}{\left(\mathrm{final}\mathrm{body}\mathrm{weight}+\mathrm{initial}\mathrm{weight}\right)}$$

$$\mathrm{Feed}\mathrm{conversion}\mathrm{ratio}\;\left(\text{FCR}\right)\;=\frac{\mathrm{feed}\mathrm{intake}}{\left(\mathrm{final}\mathrm{body}\mathrm{weight}-\mathrm{initial}\mathrm{weight}\right)}$$

$$\mathrm{Protein}\mathrm{efficiency}\mathrm{ratio}\left(\mathrm{PER}\right)\;=\frac{\left(\mathrm{final}\mathrm{body}\mathrm{weight}-\mathrm{initial}\mathrm{body}\mathrm{weight}\right)}{\mathrm{protein}\mathrm{intake}}$$

$$\mathrm{Protein}\mathrm{deposition}\mathrm{rate}\;(\mathrm{PDR},\%)=100\times \frac{\mathrm{protein}\mathrm{retention}}{\mathrm{protein}\mathrm{intake}}$$

$$\mathrm{LDR},\;\mathrm{Lipid}\mathrm{deposition}\mathrm{rate}(\%)\times 100\times \frac{\mathrm{lipid}\mathrm{retention}}{\mathrm{lipid}\mathrm{intake}}$$

$$\mathrm{Apparent}\mathrm{digestibility}\mathrm{of}\mathrm{dry}\mathrm{matter}(\%)\;=100\times \left[1-\frac{\mathrm{dietary}Y\mathrm{content}}{\mathrm{fecal}Y\mathrm{content}}\right]$$

Apparentdigestibilityofdrynutrient(%)

$$\;=100\times \left[1-\left(\frac{\mathrm{dietary}Y\text{content}}{\mathrm{fecal}Y\mathrm{content}}\right)=\left(\frac{\mathrm{dietary}Y\mathrm{content}}{\mathrm{fecal}Y\mathrm{content}}\right)\right]$$

$$\mathrm{Condition}\mathrm{factor}\left(\mathrm{CF},g/\mathrm{cm3}\right)=\frac{\mathrm{body}\mathrm{weight}}{\mathrm{body}{\mathrm{length}}^{\wedge }3}$$

$$\mathrm{Organ}\mathrm{index}\;(\mathrm{OI},\%)=100\times \frac{\mathrm{organ}\mathrm{weight}}{\mathrm{body}\mathrm{weight}}$$

$$\mathrm{Hepasomatic}\mathrm{index}\;(\mathrm{HSI},\%)=100\times \frac{\mathrm{liver}\mathrm{weight}}{\mathrm{body}\mathrm{weight}}$$

$$\mathrm{Viserosomatic}\mathrm{index}\left(\mathrm{VSI},\%\right)=100\times \frac{\mathrm{intestinal}\mathrm{weight}}{\mathrm{body}\mathrm{weight}}$$

$$\mathrm{Intestinal}\mathrm{length}\mathrm{index}\left(\mathrm{ILI},\%\right)=100\times \frac{\mathrm{intestinal}\mathrm{length}}{\mathrm{body}\mathrm{weight}}$$

### Statistical analysis

All data were subjected to one-way analysis of variance (ANOVA) with SPSS (v22.0) software, and Turkey’s multiple range test was performed when the difference was significant. *P* < 0.05 indicated that the difference between the two data was significant. Data analysis results are presented as mean ± standard error (Means ± SEM).

## Results

### Growth performance and feed utilization

**FIGURE 1:**
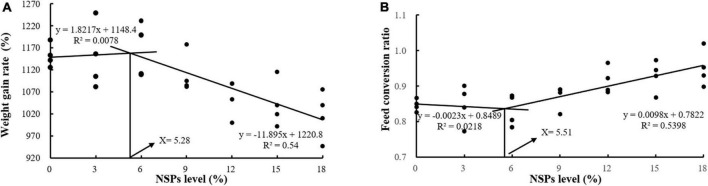
Analysis of suitable dietary non-starch polysaccharides (NSPs) levels for juvenile largemouth bass by linear regression analysis based on weight gain rate (WGR) **(A)** and feed conversion ratio (FCR) **(B)**.

Dietary inclusion of different levels of NSPs had no significant effect on the SR of juvenile largemouth bass (*P* > 0.05; Table 3). The *W*_*t*_, WGR, PER, and PDR in the NSPs12, NSPs15, and NSPs18 groups were significantly lower than those in the control group, in contrast to the FCR and FI (*P* < 0.05). In addition, the LDR in the NSPs9, NSPs12, NSP15, and NSPs18 groups was significantly lower than that in the control group (*P* < 0.05). The linear regression analysis based on WGR and FCR showed that the appropriate dietary NSPs level of juvenile largemouth bass was 5.28–5.51% (Figure 1).

**TABLE 3:** Effects of dietary inclusion of different levels of non-starch polysaccharides (NSPs) on growth performance and feed utilization of juvenile largemouth bass.

Item	Group						
	Control	NSPs3	NSPs6	NSPs9	NSPs12	NSPs15	NSPs18
Final body weight (g)	74.99 ± 0.67^a^	74.88 ± 2.28^a^	75.88 ± 1.97^a^	72.60 ± 1.38^ab^	69.49 ± 1.30^bc^	68.58 ± 1.55^bc^	67.18 ± 1.62^c^
Survival rate (%)	99.38 ± 0.63	98.13 ± 1.88	98.13 ± 1.88	99.38 ± 0.63	98.13 ± 0.63	97.50 ± 1.77	99.17 ± 0.83
Weight gain rate (%)	1151.55 ± 13.07^a^	1147.56 ± 37.25^a^	1162.48 ± 31.03^a^	1109.90 ± 22.90^ab^	1057.93 ± 20.97^b^	1041.57 ± 26.45^b^	1042.06 ± 18.93^b^
Specific growth rate (%/day)	4.51 ± 0.02^a^	4.50 ± 0.05^a^	4.53 ± 0.04^a^	4.45 ± 0.03^ab^	4.37 ± 0.03^bc^	4.35 ± 0.04^bc^	4.31 ± 0.04^c^
Feed intake (% BW/day)	2.57 ± 0.02^b^	2.58 ± 0.07^b^	2.54 ± 0.06^b^	2.63 ± 0.04^ab^	2.75 ± 0.05^a^	2.78 ± 0.06^a^	2.78 ± 0.04^a^
Feed conversion ratio	0.85 ± 0.01^b^	0.85 ± 0.03^b^	0.83 ± 0.02^b^	0.87 ± 0.02^ab^	0.92 ± 0.02^ba^	0.93 ± 0.02^ba^	0.95 ± 0.03^a^
Protein efficiency ratio	2.25 ± 0.01^a^	2.21 ± 0.04^a^	2.18 ± 0.04^a^	2.18 ± 0.03^a^	2.05 ± 0.03^b^	2.00 ± 0.01^b^	2.04 ± 0.02^b^
Protein deposition rate (%)	39.40 ± 0.40^a^	38.47 ± 1.31^a^	39.62 ± 1.08^a^	37.21 ± 1.21^a^	33.74 ± 0.69^b^	33.55 ± 0.83^b^	32.66 ± 0.87^b^
Lipid deposition rate (%)	81.34 ± 0.79^a^	81.45 ± 2.95^a^	84.25 ± 2.21^a^	73.09 ± 1.41^b^	68.45 ± 1.35^bc^	65.52 ± 1.56^c^	63.79 ± 1.64^c^

BW, body weight. Values are presented as means ± SEM (*n* = 4). In the same row, there is a significant difference between data with different superscripts (*P* < 0.05).

### Morphological parameters and chemical composition

Dietary inclusion of different levels of NSPs had no significant effect on the moisture and crude ash contents of juvenile largemouth bass (*P* > 0.05; Table 4). The crude protein and lipid contents of whole-body in the NSPs9, NSPs12, NSP15, and NSPs18 groups were significantly lower than those in the control group (*P* < 0.05). The FCR in the NSPs3 group was significantly lower than that in the control group, and the OI in the treatment group was all significantly higher than that in the control group (*P* < 0.05). The HSI in the NSPs9 group and the VSI in the NSPs15 and NSPs18 groups were significantly higher than those in the control group (*P* < 0.05). The ILI in the NSPs3 and control groups were relatively equal (*P* > 0.05), while the ILI in the rest treatment groups was significantly higher than that in the control group (*P* < 0.05).

**TABLE 4:** Effects of dietary inclusion of different levels of non-starch polysaccharides (NSPs) on morphological parameters and body composition of juvenile largemouth bass.

Item	Group						
	Control	NSPs3	NSPs6	NSPs9	NSPs12	NSPs15	NSPs18
*Morphological parameters*							
Condition factor (g/cm^3^)	2.17 ± 0.04^a^	2.04 ± 0.03^b^	2.30 ± 0.05^a^	2.23 ± 0.06^a^	2.21 ± 0.04^a^	2.15 ± 0.04^a^	2.23 ± 0.05^a^
Organ index (%)	7.55 ± 0.19^c^	8.11 ± 0.12^b^	8.05 ± 0.16^b^	8.20 ± 0.13^b^	8.11 ± 0.17^b^	8.58 ± 0.21^a^	8.36 ± 0.06^a^
Hepasomatic index (%)	1.85 ± 0.07^bc^	1.97 ± 0.07^bc^	2.01 ± 0.07^ab^	2.23 ± 0.03^a^	2.03 ± 0.08^ab^	1.98 ± 0.04^bc^	1.77 ± 0.08^c^
Viserosomatic index (%)	0.73 ± 0.02^c^	0.77 ± 0.01^c^	0.73 ± 0.03^c^	0.76 ± 0.02^c^	0.78 ± 0.02^c^	0.87 ± 0.02^b^	0.96 ± 0.03^a^
Intestinal length index (%)	0.78 ± 0.01^d^	0.82 ± 0.01^d^	0.88 ± 0.01^c^	0.89 ± 0.01^bc^	0.90 ± 0.01^ab^	0.92 ± 0.02^ab^	0.95 ± 0.02^a^
*Body composition, %*							
Moisture	72.94 ± 0.05	72.77 ± 0.33	73.02 ± 0.11	72.71 ± 0.24	72.44 ± 0.39	72.83 ± 0.33	72.62 ± 0.09
Crude protein	15.67 ± 0.06^a^	15.37 ± 0.24^a^	15.55 ± 0.15^a^	14.36 ± 0.19^b^	14.60 ± 0.15^b^	14.71 ± 0.13^b^	14.69 ± 0.17^b^
Crude lipid	8.30 ± 0.05^a^	8.14 ± 0.09^a^	8.42 ± 0.21^a^	7.69 ± 0.19^b^	7.54 ± 0.17^b^	7.36 ± 0.25^b^	7.31 ± 0.10^b^
Ash	3.62 ± 0.02	3.56 ± 0.04	3.66 ± 0.03	3.58 ± 0.03	3.55 ± 0.02	3.53 ± 0.02	3.67 ± 0.03

Values are presented as means ± SEM (*n* = 4). In the same row, there is a significant difference between data with different superscripts (*P* < 0.05).

### Nutrients apparent digestibility

The apparent digestibility of dietary crude lipids in the NSPs9 group was significantly lower than that in the control group (*P* < 0.05; Table 5). The apparent digestibility of dry matter, crude protein and lipid in the NSPs12, NSPs15, and NSPs18 groups were significantly lower than those in the control group (*P* < 0.05).

**TABLE 5:** Effects of dietary inclusion of different levels of non-starch polysaccharides (NSPs) on dietary apparent digestibility of juvenile largemouth bass (%).

Item	Group						
	Control	NSPs3	NSPs6	NSPs9	NSPs12	NSPs15	NSPs18
Dry matter	85.86 ± 0.29^a^	85.84 ± 0.18^a^	85.68 ± 0.07^a^	84.68 ± 0.16^b^	84.60 ± 0.31^b^	84.77 ± 0.07^b^	81.77 ± 0.34^c^
Crude protein	94.38 ± 0.22^a^	94.19 ± 0.25^a^	94.01 ± 0.29^a^	93.68 ± 0.12^a^	93.25 ± 0.14^b^	90.40 ± 0.25^c^	86.31 ± 0.25^d^
Crude lipid	94.50 ± 0.35^a^	94.49 ± 0.37^a^	94.42 ± 0.55^a^	91.45 ± 0.25^b^	90.45 ± 0.19^c^	90.41 ± 0.25^c^	79.34 ± 0.33^d^

Values are presented as means ± SEM (*n* = 4). In the same row, there is a significant difference between data with different superscripts (*P* < 0.05).

### Intestinal digestive and absorption enzyme activity

Dietary inclusion of different levels of NSPs had no significant effect on the activity of intestinal CK in juvenile largemouth bass (*P* < 0.05; Table 6). The activities of the intestinal protease, Na^+^/K^+^-ATPase and AKP in the NSPs12, NSPs15, and NSPs18 groups were significantly lower than those in the control group (*P* < 0.05). Additionally, the intestinal lipase activity in the NSPs15 and NSPs18 groups was significantly lower than that in the control group (*P* < 0.05). The intestinal amylase activity in the treatment group was significantly higher than that in the control group (*P* < 0.05).

**TABLE 6:** Effects of dietary inclusion of different levels of non-starch polysaccharides (NSPs) on intestinal digestive and absorptive enzyme activities of juvenile largemouth bass.

Item	Group						
	Control	NSPs3	NSPs6	NSPs9	NSPs12	NSPs15	NSPs18
Protease (U/g protein)	4.47 ± 0.39^a^	4.40 ± 0.26^a^	4.73 ± 0.34^a^	4.31 ± 0.21^a^	3.57 ± 0.35^b^	3.71 ± 0.20^b^	3.43 ± 0.24^b^
Lipase (U/g protein)	0.96 ± 0.05^a^	1.02 ± 0.03^a^	1.01 ± 0.03^a^	0.90 ± 0.07^a^	0.93 ± 0.08^a^	0.83 ± 0.03^b^	0.78 ± 0.05^b^
Amylase (IU/g protein)	0.25 ± 0.03^b^	0.32 ± 0.03^a^	0.40 ± 0.04^a^	0.33 ± 0.03^a^	0.35 ± 0.03^a^	0.32 ± 0.02^a^	0.34 ± 0.03^a^
Creatine kinase (U/mg protein)	0.15 ± 0.02	0.14 ± 0.02	0.16 ± 0.02	0.17 ± 0.02	0.15 ± 0.01	0.15 ± 0.01	0.13 ± 0.01
Na^+^/K^+^-ATPase (U/mg protein)	23.50 ± 0.42^a^	24.08 ± 1.04^a^	23.88 ± 0.65^a^	22.38 ± 1.17^ab^	21.44 ± 1.28^b^	21.47 ± 0.30^b^	20.76 ± 0.43^b^
Alkaline phosphatase (U/g protein)	208.14 ± 12.07^a^	196.88 ± 9.34^a^	225.69 ± 8.08^a^	204.59 ± 7.47^a^	169.47 ± 11.54^b^	155.97 ± 6.79^b^	1255.88 ± 4.60^b^

Values are presented as means ± SEM (*n* = 4). In the same row, there is a significant difference between data with different superscripts (*P* < 0.05).

### Morphological observation of hindgut

**FIGURE 2:**
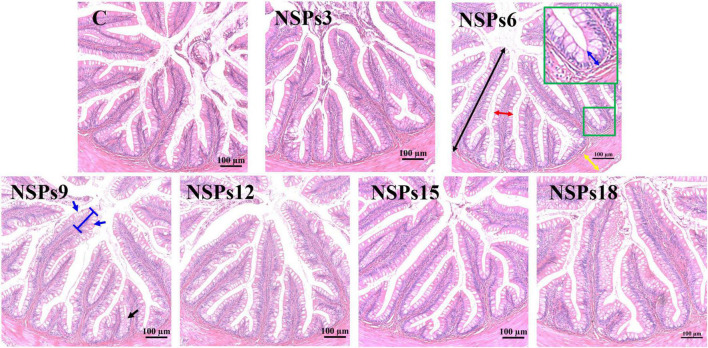
Effects of different non-starch polysaccharides (NSPs) levels on hindgut morphology of juvenile largemouth bass (H&E staining, magnification × 200). Black double-sided arrow: villus height; red double-sided arrow: villus width; blue double-sided arrow: crypt depth; yellow double-sided arrow: muscularis thickness; blue arrow: goblet cell.

**FIGURE 3:**
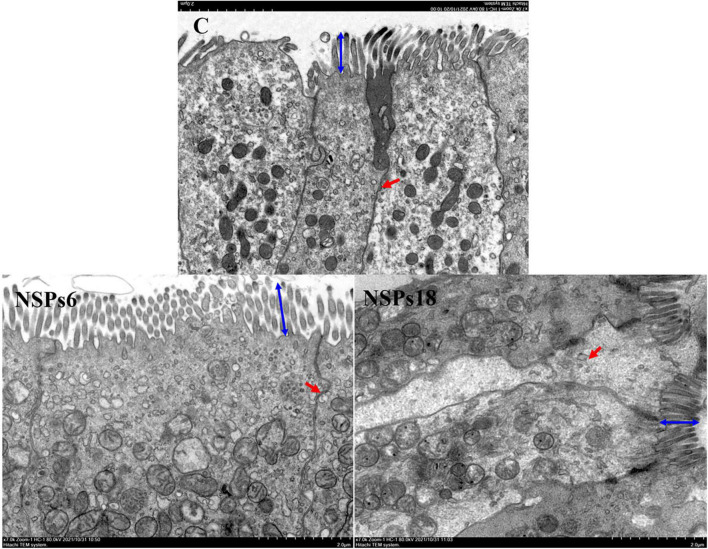
Effects of different non-starch polysaccharides (NSPs) levels on hindgut morphology of juvenile largemouth bass (transmission electron microscope observation, magnification × 7,000). Blue double-sided arrow: microvillus height; red arrow: epithelial cell interval.

**FIGURE 4:**
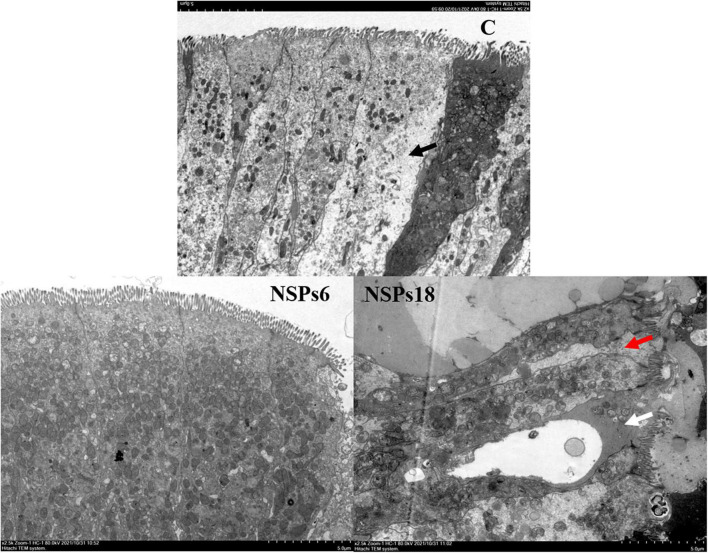
Effects of different levels of dietary non-starch polysaccharides (NSPs) on hindgut morphology of juvenile largemouth bass (transmission electron microscope observation, magnification × 2,500). Red arrow: epithelial cell interval; black arrow: epithelial cell abnormalities; white arrow: epithelial cell death.

Morphological observations of the hindgut are shown in Figures 2, 3 where the measurements are also labeled. Dietary inclusion of different levels of NSPs had no significant effect on the length of the intestinal microvillus in the hindgut of juvenile largemouth bass (*P* < 0.05; Table 7). The height of hindgut villus in the NSPs15 and NSPs18 groups was significantly higher than that in the control group (*P* < 0.05). The width of hindgut villus in the NSPs9, NSPs12, NSPs15, and NSPs18 groups was significantly higher than that in the control group, in contrast to the crypt depth (*P* < 0.05). The muscularis thickness of hindgut in the NSPs12, NSPs15, and NSPs18 groups was significantly higher than that in the control group (*P* < 0.05). Except for the NSPs3 group, the relative goblet cell numbers of hindgut villus in the rest treatment groups were significantly higher than that in the control group (*P* < 0.05). Intestinal epithelial cell death was observed in the NSPs18 group (Figure 4).

**TABLE 7:** Effects of dietary inclusion of different levels of non-starch polysaccharides (NSPs) on hindgut morphology of juvenile largemouth bass.

Item	Group						
	Control	NSPs3	NSPs6	NSPs9	NSPs12	NSPs15	NSPs18
Villus height (μm)	465.56 ± 18.88^c^	524.93 ± 24.84^bc^	544.40 ± 13.29^bc^	514.12 ± 18.77^bc^	529.20 ± 19.57^bc^	575.56 ± 27.48^b^	649.11 ± 30.08^a^
Villus width (μm)	87.11 ± 1.26^c^	91.64 ± 3.36^bc^	91.65 ± 3.25^bc^	96.29 ± 1.70^b^	100.52 ± 3.01^b^	98.29 ± 3.65^b^	107.67 ± 4.54^a^
crypt depth (μm)	28.06 ± 1.03^a^	28.12 ± 1.74^a^	27.87 ± 1.89^a^	22.69 ± 1.91^b^	23.69 ± 1.45^b^	23.78 ± 0.45^b^	24.13 ± 1.26^b^
Muscularis thickness (μm)	96.30 ± 8.25^b^	95.15 ± 10.24^b^	96.57 ± 7.03^b^	98.62 ± 10.08^b^	136.03 ± 17.01^a^	143.33 ± 8.61^a^	148.67 ± 3.10^a^
Goblet cell relative number (per 100 μm)	12.17 ± 0.70^d^	12.00 ± 0.52^d^	14.50 ± 0.96^c^	17.00 ± 0.63^b^	17.00 ± 0.62^b^	17.60 ± 0.90^b^	19.75 ± 0.85^a^
Microvillus height (μm)	0.92 ± 0.03	−	0.96 ± 0.04	−	−	−	0.92 ± 0.02

Values are presented as means ± SEM (*n* = 4). In the same row, there is a significant difference between data with different superscripts (*P* < 0.05).

### Gene expression parameters of hindgut

**FIGURE 5:**
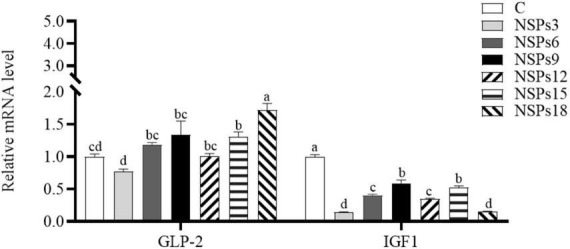
Effects of different levels of dietary non-starch polysaccharides (NSPs) on intestinal insulin-like growth factor 1 (IGF1) and glucagon-like peptide 2 (GLP-2) expression levels in juvenile largemouth bass. Bars represent the mean ± SEM (*n* = 4), and different superscript letters represent significant differences between treatments (*P* < 0.05).

The expression level of the IGF1 in the NSPs groups was dramatically lower than in the control group, whereas GLP-2 gene’s expression in the NSPs15 and NSPs18 groups was dramatically higher than that in the control group (*P* < 0.05; Figure 5).

## Discussion

Generally, dietary NSPs are also considered dietary fiber (28), and are considered as dietary components of no or little nutritional value in fish diets (12). Although dietary fiber cannot be directly utilized by fish, it can still have varying degrees of physiological effects on fish (1). Some studies have shown that low dietary fiber diets benefit fish growth, while high dietary fiber diets inhibit fish growth (22, 29, 30). Therefore, NRC (31) recommends that fish dietary fiber levels should not exceed 10%. Due to the complex and diverse composition of NSPs and the immature extraction process, there are relatively few studies on dietary NSPs in fish. In this study, the low NSPs diet (<9%) had no adverse effect on the growth of juvenile largemouth bass, while the high NSPs diet (>9%) significantly inhibited the growth of juvenile largemouth bass. Similarly, Deng et al. (12) also found that high NSPs (24.8% NSPs) diets significantly inhibited the growth performance of rainbow trout (*Oncorhynchus mykiss*). Conversely, several studies have found that the dietary inclusion of high NSPs had no negative effects on the growth performance of rainbow trout (15% cellulose) and European sea bass (*Dicentrarchus labrax* L., 15.5% cellulose) (32, 33). The differences in these results suggest that the effect of dietary NSPs on fish growth may depend on the composition of dietary NSPs and the species of fish tested.

The results of the linear regression analysis based on the weight gain rate and feed coefficient of the test fish showed that the level of dietary NSPs suitable for the growth of juvenile largemouth bass was 5.28–5.51%, and this value is slightly higher than the result reported by Shi (34) that the optimum dietary fiber level for largemouth bass (21.64 ± 0.12 g) was 4.07%. The differences in the two results may be related to factors such as the size of the experimental fish, the nutritional level of the feed, and the difference in the composition of dietary NSPs and dietary fiber (dietary fiber contains both dietary NSPs and lignin). This also reflects the difference in the tolerance of fish to dietary NSPs and dietary fiber. Furthermore, this value is far lower than the optimum fiber level of the Jian carp (9.19%) and grass carp (14.39–16.42%) (*Ctenopharyngodon idella*) (22, 35). The differences in these results fully indicate that carnivorous fish are far less tolerant of dietary fiber than herbivorous and omnivorous fish. Intestinal digestive enzymes are essential in the absorption of feed nutrient in fish, and their activity determines the nutrients absorption efficiency and growth rate of fish (36–38). Meanwhile, the characteristics and quantities of feed ingredients also affect the activity of digestive enzymes (39, 40). In this study, the activities of the intestinal protease, lipase, Na^+^/K^+^-ATPase and CK of largemouth bass juveniles under the high NSPs diet were significantly decreased, which was highly consistent with the growth performance of largemouth bass juveniles. This phenomenon indicates that the poor growth of fish caused by a high NSPs diet may be related to the inhibitory effect of dietary NSPs on the activities of intestinal digestion and absorption enzymes. Moreover, AKP is not only directly involved in the transfer and metabolism of phosphate units, but also considered as an important immune enzyme in fish, and its activity can reflect the immunity status of the fish organism (41, 42). Thus, it can be speculated that dietary inclusion of high levels of NSPs was detrimental to the intestinal health of juvenile largemouth bass in this study. Also, the observation of intestinal epithelial cell death in the NSPs18 group confirmed this hypothesis.

Dietary protein and lipid need to be decomposed by protease and lipase before they can be absorbed and utilized by the intestinal tract. Consequently, the apparent digestibility of dietary protein and lipid in fish was linearly correlated with the activity of intestinal digestive enzymes (43). In this study, diet inclusion of high levels of NSPs significantly reduced the apparent digestibility of feed dry matter, crude protein, and lipid, which was consistent with the changes in intestinal digestion and absorption enzyme activities. Therefore, it can be speculated that the significant decrease in the apparent digestibility of feed nutrients caused by the high NSPs diet in this study may be related to the decrease in the activities of intestinal digestion and absorption enzymes. Further, the decrease in digestive enzyme activity and apparent digestibility of feed nutrients ultimately caused reduced crude protein and lipid contents of the whole-body, as well as the PER, PDR, and LDR of juvenile largemouth bass. Meanwhile, some studies have suggested that dietary NSPs stimulate the secretion of mucus in the gut, thereby accelerating endogenous nitrogen loss (44, 45). Rgensen et al. (46) suggested that endogenous nitrogen loss caused by dietary NSPs is also one of the reasons for the decrease in apparent protein digestibility. Thus, dietary inclusion of high levels of NSPs leading to a significant reduction in the apparent digestibility of crude protein may also be associated with endogenous nitrogen loss.

The effects of dietary NSPs on the activity of intestinal digestive enzymes may be based on the physicochemical properties of NSPs. For instance, some soluble NSPs (SNSPs, e.g., arabinoxylan, mannans, and β-glucan) are naturally in viscous and can increase the viscosity of chyme and adhere to digestive enzymes, thereby inhibiting digestive enzymes (47); meanwhile, the adhesion of sticky granules to intestinal villus may also negatively affect the activity of digestive enzymes (48). In addition, the complexation of dietary NSPs with digestive enzymes or the encapsulation of substrates may also cause a decrease in digestive enzyme activity (1). Deng et al. (12) found that dietary inclusion of 16.8% SNSP extremely decreased the intestinal protease activity and growth performance of rainbow trout compared to inclusion of 24.8% NSPs. Therefore, it can be considered that the decrease of digestive enzyme activity caused by dietary NSPs may be related to SNSP components.

There are a large number of carboxyl and hydroxyl units that can interact with mineral elements in the chemical structure of polysaccharides that constitute dietary NSPs (49). The mineral elements bound with these functional units may cause the efflux of mineral elements from the intestine with dietary NSPs, which in turn reduces the concentration of mineral elements in the intestine and their absorption efficiency. Studies on fish found that diets rich in NSPs significantly reduced the uptake efficiency of dietary sodium (Na) in Nile tilapia (*Oreochromis niloticus* L.) and calcium, magnesium, phosphorus (P) and Na in African catfish (*Clarias gariepinus*) (17, 50). Similarly, dietary supplementation with defatted soybean meal significantly inhibited the absorption of zinc, Na, and kalium (K) in Atlantic salmon, and fecal excretion of copper, ferrum, Na, and K were significantly increased when dietary supplementation with soybean NSPs (51); and increasing the concentration of dietary NSPs (cellulose) significantly increased fecal excretion of Na in rainbow trout (32). These results suggest that dietary NSPs may reduce the concentration of mineral elements (especially Na) in the fish gut, which may reduce the osmotic pressure in the gut content. Na^+^/K^+^-ATPase is a membrane enzyme that can transport K^+^ into and Na^+^ out of cells in reverse concentrations, and plays an important role in biological processes such as maintaining cellular osmotic pressure and nutrient uptake (52, 53). The activity of Na^+^/K^+^-ATPase is usually affected by the osmotic pressure inside and outside the cell membrane (54), and is closely related to substrate ion concentration (53, 55). Gal-Garber et al. (53) have found that reducing the concentration of Na in the diet can significantly reduce the activity of Na^+^/K^+^-ATPase in the small intestine of chickens, but lead to a compensatory increase in the expression level of Na^+^/K^+^-ATPase gene. Thus, it can be hypothesized that the decrease of intestinal Na^+^/K^+^-ATPase caused by high levels of dietary NSPs in this study may be associated with the efflux of Na, P and other mineral elements with NSPs, but this hypothesis needs further testing.

Feed ingredients inevitably affect the development and morphology of the intestine, therefore, intestinal morphology is often used to assess the potential physiological effects of diet on the intestine (56). Moreover, the morphology of the fish intestine is closely related to its physiological functions such as digestion and absorption (57–59). For instance, changes in the number of intestinal folds and goblet cells, the height and distribution of villus may affect intestinal digestive and absorption functions (38, 60–62). It is generally believed that the factors that can increase the digestive area of the intestine can improve the digestion and absorption of feed nutrients in the intestine. In this study, dietary inclusion of high levels of NSPs significantly altered the intestinal morphology of juvenile largemouth bass, including the increase in the length and width of intestinal villus, muscularis thickness and the number of goblet cells, and decrease in crypt depth. The increase in the length and width of the intestinal villus and decrease in crypt depth imply a larger intestinal absorption area, while the increase in muscularis thickness indicates that the intestinal peristalsis is enhanced, all these changes are beneficial to the digestion and absorption of feed nutrients (63, 64). Furthermore, increased villus length also implies increased turnover of intestinal epithelial cells (65). This evidence suggests that dietary inclusion of high levels of NSPs enhanced the intestinal absorption capacity of juvenile largemouth bass in this study, however, this is in contrast to the results of decreased growth performance, apparent nutrient digestibility, and activity of intestinal digestion and absorption enzymes. Therefore, it can be speculated that the changes in intestinal morphology are an adaptation of fish to high levels of dietary NSPs, thereby resisting the reduced nutrient intake caused by high levels of dietary NSPs.

Mucin secreted by goblet cells is an important component of the immune barrier of the intestinal mucosa and plays an important role in the maintenance of the intestinal health of fish (66–68). Therefore, an increase in the number of goblet cells is generally considered beneficial in promoting intestinal health. However, Wang et al. (69) concluded that an excessive increase in the number of goblet cells will thicken the mucus layer, which is detrimental to the absorption of feed nutrients in the intestine. In addition, some studies have found that increasing dietary NSPs or fiber levels results in the efflux of mucus from the intestinal lumen with the chyme in monogastric animals (70–72), accompanied by an increase in the number of intestinal goblet cells (72). Sinha et al. (1) suggest that dietary NSPs increase the volume of chyme and physically scrape the intestinal mucus layer as it passes through the intestine, leading to mucus efflux. This evidence suggests that the increase in the number of intestinal goblet cells caused by high levels of dietary NSPs may be associated with intestinal mucus efflux, which requires more goblet cells to secrete mucus to keep the intestinal mucus layer complete. This may also be a repair strategy for the intestine to cope with damage to the mucosal layer, implying that high NSPs are detrimental to fish intestinal health.

Studies have revealed that IGF1 and GLP-2 play an important role in regulating nutrient metabolism and promoting cell proliferation and differentiation, and their expression levels can effectively reflect the development status of tissues (73–75). Additionally, these gene’s expression level can be regulated by dietary ingredients (76, 77). In this study, dietary NSPs inclusion level above 15% significantly increased intestinal GLP-2 gene’s expression, indicating that high levels of dietary NSPs were beneficial to intestinal development, which may explain the significant increase in VSI and ILI of juvenile largemouth bass. Based on the fact that excessive NSPs have adverse effects on fish digestion and absorption and growth, digestive tract lengthening and weighting may also be strategies for fish to cope with the negative effects of dietary NSPs. However, the mechanisms by which dietary NSPs up-regulate GLP-2 gene’s expression and increase intestinal length and weight are still unclear and need to be further studied.

## Conclusion

In summary, using weight gain rate and feed coefficient as evaluation indicators, the suitable level of NSPs for largemouth bass juveniles should not above 5.51%. Excessive dietary NSPs adversely affected digestive and absorptive enzyme activities and intestinal morphology, which in turn reduced the apparent digestibility of feed nutrients and the growth of juvenile largemouth bass. Overall, our data suggest that carnivorous fish have limited tolerance to dietary NSPs, and for juvenile largemouth bass, dietary NSPs should not exceed 5.51%.

## Data availability statement

The original contributions presented in this study are included in the article/Supplementary material, further inquiries can be directed to the corresponding authors.

## Ethics statement

This animal study was reviewed and approved by Animal Research and Ethics Committee of Guangdong Ocean University.

## Author contributions

YL: conceptualization, formal analysis, data curation, writing—original draft, and writing—review and editing. JF, HH, and HZ: methodology, project administration, and data curation. YC and YZ: conceptualization, formal analysis, and data curation. WJ and WZ: project administration and supervision. JD and BT: investigation, methodology, and resources. All authors contributed to the article and approved the submitted version.
